# Heat-sensitive moxibustion for anaphylactic rhinitis: A protocol of systematic review and meta-analysis of randomized clinical trials

**DOI:** 10.1097/MD.0000000000018557

**Published:** 2020-01-24

**Authors:** Jun Xiong, Jun Yang, Ting Yuan, Xue Wang, Yunfeng Jiang, Xiaohong Zhou, Kai Liao, Lingling Xu

**Affiliations:** a Affiliated Hospital of Jiangxi University of TCM; b Jiangxi University of TCM, Nanchang, Jiangxi, PR China.

**Keywords:** anaphylactic rhinitis, heat sensitive moxibustion, protocol, systematic review

## Abstract

**Background:**

Anaphylactic rhinitis (AR) is one of the most common allergic disorders globally. Heat-sensitive moxibustion (HSM) is an effective method for AR without the occurrence of drug damage. This study aims to systematically investigate the effectiveness and safety of HSM for patients with AR.

**Methods:**

Seven relevant electronic databases from inception until January 2020 including PubMed, Embase, Cochrane Library, the China National Knowledge Infrastructure Database, Wanfang Database, Chinese Biomedical Literature Database, and Chinese Scientific Journal Database will be searched. All relevant randomized clinical trials published in English and Chinese about HSM for AR regardless of blinding or publication types can be included. The Cochrane Central Register of Controlled Trials and other potential articles in the reference list of included studies will also be searched. We recommend total nasal symptom score as primary outcomes. Secondary outcomes includes rhinoconjunctivitis quality of life questionnaire, IgE, visual analogue scale, laboratory examination, and side effects. Study selection, data extraction, and quality assessment will be performed independently by 2 reviewers. Assessment of risk of bias and data synthesis will be conducted by RevMan 5.3 software.

**Ethics and dissemination:**

Ethical approval is not required for no personal data involved. The results of this SR will be disseminated through peer-reviewed publications according to the Preferred Reporting Items for Systematic reviews and Meta Analysis Protocols guidelines.

**Results:**

The results will be published in a peer-reviewed journal.

**Conclusion:**

The findings will provide further evidence for the management of AR.

**PROSPERO registration number:**

CRD42019140723.

## 1 Introduction

### 1.1 Description of the condition

Anaphylactic rhinitis (AR) is a prevalent noninfectious inflammatory disorder of the nasal mucosa, which mainly triggers IgE response after people being exposed to different allergens. Clinically, nasal obstruction, frequent sneezing, runny nose, itchy and watery eyes are the main manifestations, accompanying with smell sense losing.^[1,2]^ In some case, this condition severely affects the quality of people's life.^[3–5]^

People of all ages may get AR, whereas approximately 80% of individuals diagnosed with allergic rhinitis develop symptoms before the age of 20 years. Thirteen percent of US children were diagnosed as having AR^[6]^ and 15.79% in China.^[7]^ Ten years ago, 40% world's population were affected^[8,9]^ and the prevalence of AR in Europe were 23% to 30%.^[10]^ A survey conducted in 2005 has reported that almost 800 million people suffered from the disease in the rural areas of China.^[7]^ Nowadays, the prevalence of AR rises annually across many countries.^[3]^

The economic survey has showed that they spent more money on medication costs and health care in a family with AR patients.^[11]^ There is no doubt that extra expenses will increase the financial burden on the family. In the meantime, AR will increase the risk of other diseases such as headaches, upper airway cough syndrome, hypomnesis, chronic rhinosinusitis, and asthma. It is worth noting that AR is an independent risk factor for the development of asthma. According to a survey conducted in the United States, 78% of asthma patients have AR.^[12]^

According to the occurrence frequency, AR can be divided into intermittent (<4 days for 1 week or <4 weeks for a year) and persistent (>4 days for 1 week and >4 weeks for a year).^[8]^ The therapeutic principle of AR involves a comprehensive approach including environmental control, pharmacotherapy, immunotherapy, and patient education.^[8]^

Antihistamines^[13,14]^ and intranasal corticosteroid spray^[15]^ as the first-line therapy for AR works through blocking multiple inflammatory pathways to alleviate the painful sufferings and nasal symptoms; however, recurrence of the disease makes medication less effective over time. Intranasal anticholinergics are recommended as second-line therapy for AR by inhibiting both watery secretion of nasal glands and vasodilatation of airway blood vessels.^[16]^ Based on the theory of traditional Chinese medicine, some Chinese herbal formulae such as Yu-ping-feng San, Xiao Qing Long decoction, Bu Zhong Yi Qi decoction, Shenling Baizhu decoction, and so on, were proved effective and safe in improving the nasal symptoms of perennial and persistent AR through adjusting cyclic nucleotide levels in the body, inhibiting IgE and mast cell degranulation, and restoring blood flow in nasal mucosa.^[17–20]^

### 1.2 Description of the intervention

HSM therapy, as one of suspended moxibustion therapies,^[21]^ can effectively improve the symptoms of AR patients in practice. HSM refers to practitioners administer moxibustion on heat-sensitive acupuncture points, which are extremely sensitive to the heat stimulation of burning moxa.^[22]^

The therapy has dual-directional regulation effects on human's immune system to balance our inner environment and function, which could improve the blood indices with an increased volume of blood flow and regulate the immunologic function of the human body which brings out therapeutic effects for AR.^[23]^ The latest American Clinical Practice Guideline for AR published in 2015 recommends that acupuncture be offered as an option for patients with interest in nonpharmacologic approaches to management of AR.^[24,25]^

Systematic review or meta analysis has been recognized as the basis of evaluating clinical efficacy and formulating clinical guidelines.^[26,27]^ There is 1 systematic review about the effectiveness of HSM for AR published in Chinese.^[28]^ The previous review included 7 RCTs; it does not refer to other outcomes such as adverse events, recurrence rate, quality of life, and so on. It also did not provide study protocols in advance. Due to the limitations of previous study, the results may uncertain. Therefore, we take stricter search strategy and objective outcome evaluation to conduct this systematic review, to provide reliable evidence about the effectiveness and safety of HSM for AR.

## 2 Methods and analysis

### 2.1 Study registration

This systematic review protocol has been registered on PROSPERO as CRD42019140723. which was available from: https://www.crd.york.ac.uk/PROSPERO/display_record.php?RecordID=140723. The protocol follows the Cochrane Handbook for Systematic Reviews and Meta-Analysis Protocol (PRISMA-P) statement guidelines.^[29]^ We will describe the changes in our full review if needed.

### 2.2 Inclusion criteria

#### 2.2.1 Type of studies

All relevant randomized clinical trials (RCTs) published in English and Chinese about HSM for AR regardless of blinding or publication types can be included.

#### 2.2.2 Type of participants

Study participants in different age ranges with all types of AR can be included, regardless of sex, race, occupation, education, nationality, etiology, severity.

#### 2.2.3 Type of interventions

HSM as a single intervention or major part of a combination therapy with other active intervention (eg, conventional drugs, acupuncture, Chinese herbs, and so on) will be included.

#### 2.2.4 Type of comparators

The comparative interventions could be sham moxibustion, placebo, no treatment, or other active treatments. The following treatment comparisons will be selected.

1. Heat-sensitive moxibustion (HSM) versus no treatment.
2. HSM versus placebo or sham acupuncture.
3. HSM versus other active therapies.
4. HSM + active therapy versus the same active therapy.

#### 2.2.5 Types of outcome measurements

##### 2.2.5.1 Primary outcomes

We recommend the total nasal symptom score^[30]^ and as the primary outcome and evaluate the sore according to the degree of 4 nasal symptoms: sneezing, rhinorrhea, nasal itching and nasal obstruction. We will also compare the score change before and after treatment, at least a treatment cycle.

##### 2.2.5.2 Secondary outcomes

The secondary outcomes of this review mainly include the following aspects:

1. Rhinoconjunctivitis quality of life questionnaire.^[31]^
2. Allergen detection: IgE.
3. Side effects and adverse events.
4. VAS: Patients grade their symptoms by putting a vertical line on a 1- to 10-cm line representing severity score from 0 “no symptoms” to 10 “highest level of symptoms."
5. Laboratory examination: IgA, IgM, IgG, C3, C4.

### 2.3 Exclusion criteria

The exclusion criteria of this review mainly include the following aspects:

1. Patients with asthma, nasosinusitis, vasomotor rhinitis, infectious rhinitis, hormonal rhinitis were excluded.
2. Repeated publications and no test data required by this program are available.
3. Full text cannot be obtained through various approaches.

### 2.4 Search methods for identification of studies

#### 2.4.1 Electronic searches

Seven relevant electronic databases from inception until January 2020 including PubMed, Embase, Cochrane Library, the China National Knowledge Infrastructure Database, Wanfang Database, Chinese Biomedical Literature Database nd Chinese Scientific Journal Database will be searched. The language within in Chinese and English. The following search terms will be used: thermal moxibustion, heat-sensitive acupoint, HSM, allergic rhinitis, AR, nasal allergy, perennial allergic rhinitis, seasonal allergic rhinitis, hay fever, pollinosis. The search strategy for PubMed is shown in Table 1.

**Table 1:**
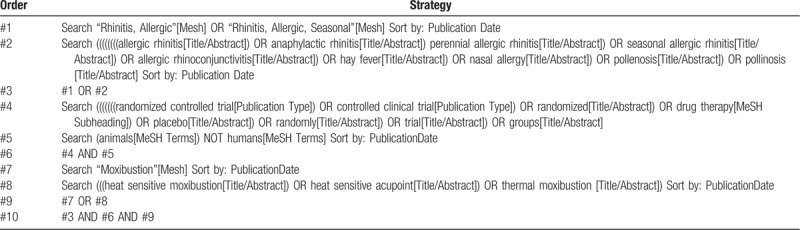
Search Strategy (PubMed).

#### 2.4.2 Additional search

To make sure comprehensiveness of the study, we will search the Cochrane Central Register of Controlled Trials and other potential articles in the reference list of included studies.

### 2.5 Studies selection

**Figure 1:**
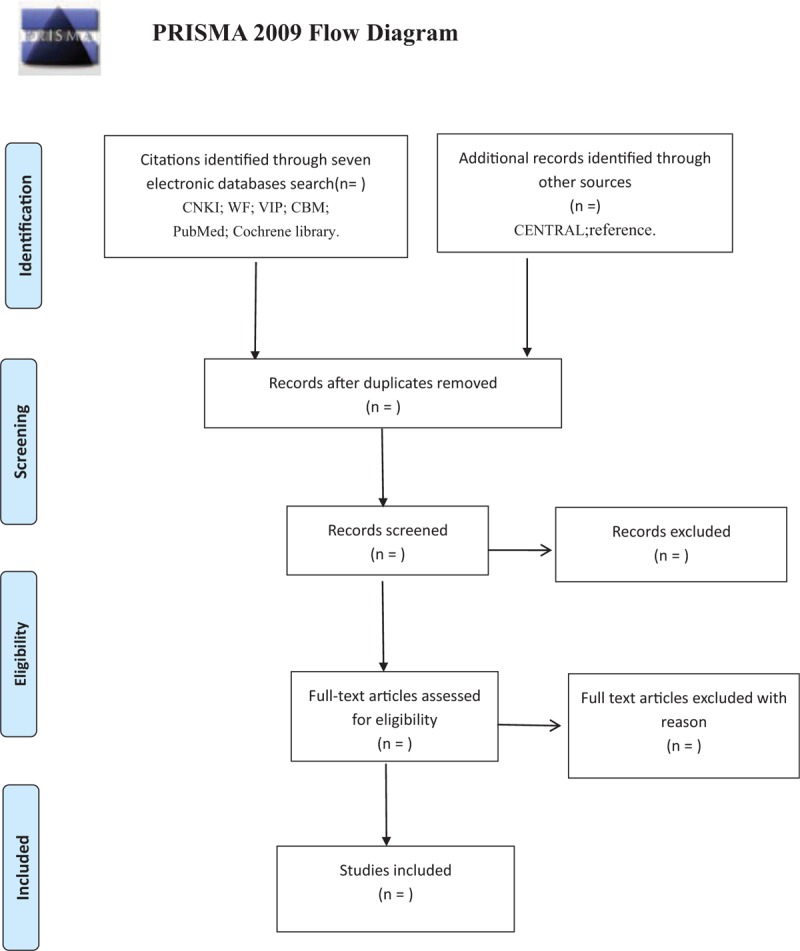
Flowchart of literature selection.

All the retrieved studies will be managed with NoteExpress 3.0, and the duplicated studies will be discarded. According to the inclusion criteria, 2 reviewers (TY and XW) separately discard some articles that are not eligible by looking through the titles and abstracts. Then they download the full texts of all possibly relevant studies for further check to make the final decision. If articles contain insufficient information to make a decision about eligibility, JY will try to contact authors of the original reports to obtain further details. During the procedure, disagreements will be resolved by discussion or consensus with the third reviewer (FYJ). The procedures of study selection will be performed in accordance with the Preferred Reporting Items for Systematic reviews and Meta Analysis^[32]^ flowchart (Fig. 1).

### 2.6 Data extraction

Two reviewers (XHZ and LLX) will independently extract the following information: author, publish year, participants, intervention (s), comparison (s), outcome (s), and some relevant characteristics from the full-text. If the reported data are not sufficient, JY will try to contact the authors for further information to supplement the missing data. Any disagreements will be resolved by discussion or consensus with the third reviewer (KL).

### 2.7 Data analysis

#### 2.7.1 Assessment of risk of bias and reporting quality of included studies

Two reviewers (TY and XW) will independently assess the methodological quality of included studies on the basis of the bias risk assessment tool of the Cochrane Collaborative Network System Evaluator’ Manual for Systematic Reviews of Interventions.^[33]^ Researcher evaluates 7 items (random sequence generation, allocation concealment, Blinding of participants, personnel Blinding of outcome assessment, incomplete outcome data, selective outcome reporting and other bias) of the methodological characteristics in high, low, unclear. Meanwhile, the Consolidated Standards of Reporting Trials ^[34]^ and Standards for Reporting Interventions in Clinical Trials of Moxibustion^[35]^ checklist will be also conducted. During the process, disagreement will be solved by communication or consulting for a third reviewer (JX) until the result comes to an agreement.

#### 2.7.2 Measures of treatment effect

In our study, we will use RevMan 5.3 software to conduct statistical analyses. The dichotomous data will be analyzed by risk ratio with 95% confidence intervals (CIs), and the continuous data will be analyzed by mean difference (MD) or standard MD with 95% CIs. When meta-analysis is not possible, we will perform narrative analysis.

#### 2.7.3 Unit of analysis issues

We will only select the first-phase data of crossover trials to avoid carryover effects. When it comes to multiple intervention groups, we will combine all relevant experimental groups and control intervention groups into a single respectively group to prevent a unit of analysis issue.

#### 2.7.4 Dealing with missing data

When the data provided are insufficient, we will attempt to contact the original authors to obtain the missing information. If the missing data are not available, the analysis will rely on available data.

#### 2.7.5 Assessment of heterogeneity

We choose *I*^*2*^ test calculate by RevMan5.3.5 software to assess the heterogeneity of included studies.^[36,37]^ According to the heterogeneity levels of the included studies, the random-effects model (*I*^*2*^ ≥50%) or fixed-effects model (*I*^*2*^ *<* 50%) will be properly selected. If the *I*^*2*^ test is >75%, we should find possible reasons from clinical and methodological aspects by conducting subgroup or sensitivity analysis.

#### 2.7.6 Subgroup analysis

If necessary, subgroup analysis will be conducted according to the age, sex, type of AR, treatment frequency, length of follow-up.

#### 2.7.7 Sensitivity analysis

If there is high heterogeneity (the *I*^*2*^ test is >75%) in our study, we will perform sensitivity analyses from sample size, methodological quality, and characteristic of research. Then we will obtain a stable consolidated result of our study.

#### 2.7.8 Publication bias

Publication bias will be assessed by funnel plot when the included studies >10.

#### 2.7.9 Grading of Recommendations Assessment

The quality of evidence will be evaluated by the Grading of Recommendations Assessment, Development, and Evaluation system.^[38]^ The evidence quality of all outcomes will be assessed by 4 levels (high, moderate, low, or very low) from 5 aspects (limitations, inconsistency, indirectness, imprecision, and publication bias).^[39]^

#### 2.7.10 Ethics and dissemination

Ethical approval is not required for no personal data involved. The results of this SR will be disseminated through peer-reviewed publications according to the PRISMA-P guidelines.

## 3 Discussion

AR is one of the most common allergic disorders globally and it affects 10% to 40% of the world's population.^[40]^ Many people suffer from the negative influence of AR, so patients may resort medicine to relive the uncomfortable symptoms. However, the most common adverse events of intranasal formulations usage including nasal burning, poor taste, sedation, more frequent dosing and increased cost relative to oral formulations. Doctors should pay more attention to side effects with the initiation of intranasal antihistamines.

In Traditional Chinese Medicine, AR belongs to the category of “Bi Qiu," the cause of AR was considered from deficiency, excess, cold or heat, involving the “Zang-Fu," organs of the lungs, spleen, and kidneys at the basis of traditional Chinese medicine theory. There are many methods to help patients improve their uncomfortable symptoms.

HSM, as one of effective therapy for AR, is derived from some ancient Chinese medical literatures such as the Yellow Emperor's Internal Classics more than 2500 years ago.^[41]^ Unlike common suspended moxibustion at fixed acupuncture points, this therapy believed that the responsive site (s) may dynamically change with disease progression raises.^[42]^ Therefore, this technique emphasizes the presence or absence of heat sensitization responses from patients on the treatment. It is reported that the occurrence of heat sensitization appears 10% in healthy people and 70% in sick people. These responses gradually disappear with disease recovery.^[21,43]^ In large clinical practices, researchers have summarized several types of heat sensitization responses as follows: heat penetration, heat expansion, heat transmission, and nonthermal sensations (such as perceive aching, heaviness, pain, numbness, pressure, or cold in local or distant locations of stimulation). Those heat sensitization responses may appear alone or in combination.^[42]^

HSM can increase blood volume, speed the blood flow, and regulate the immunologic function of the patients with AR. Nowadays, increasing number of original articles and clinical trials have reported the effectiveness and safety of HSM for patients with AR. There are some limitations in the previous studies, without providing study protocols and absence of reporting about adverse events, quality of life and so on. Therefore, we conduct this systematic review to assess the effectiveness and safety of HSM for patients with AR. We hope our study could help establish a better approach for the treatment of AR and provide reliable evidence.

## Author contributions

**Conceptualization:** Jun Xiong, Jun Yang , Ting Yuan.

**Data curation:** Jun Yang, Ting Yuan, Xue Wang.

**Formal analysis:** Yunfeng Jiang, Xiaohong Zhou, Lingling Xu.

**Investigation:** Jun Xiong, Jun Yang.

**Methodology:** Jun Xiong, Ting Yuan, Xue Wang.

**Software:** Yunfeng Jiang, Xiaohong Zhou, Lingling Xu.

**Supervision:** Jun Xiong, Kai Liao.

**Writing – original draft:** Jun Xiong, Jun Yang, Ting Yuan, Xue Wang.

**Writing – review & editing:** Yunfeng Jiang, Xiaohong Zhou, Kai Liao, Lingling Xu.

Jun Yang orcid: 0000-0002-2804-2267.
